# Xentry, a new class of cell-penetrating peptide uniquely equipped for delivery of drugs

**DOI:** 10.1038/srep01661

**Published:** 2013-04-16

**Authors:** Kristopher Montrose, Yi Yang, Xueying Sun, Siouxsie Wiles, Geoffrey W. Krissansen

**Affiliations:** 1 Department of Molecular Medicine & Pathology, Faculty of Medical and Health Sciences, University of Auckland, Auckland 1005, New Zealand

## Abstract

Here we describe an entirely new class of cell-penetrating peptide (CPP) represented by the short peptide Xentry (LCLRPVG) derived from an N-terminal region of the X-protein of the hepatitis B virus. Xentry permeates adherent cells using syndecan-4 as a portal for entry, and is uniquely restricted from entering syndecan-deficient, non-adherent cells, such as resting blood cells. Intravenous injection of Xentry alone or conjugated to β-galactosidase led to its delivery to most tissues in mice, except circulating blood cells. There was a predilection for uptake by epithelia. Anti-B-raf antibodies and siRNAs linked to Xentry were capable of killing B-raf-dependent melanoma cells. Xentry represents a new class of CPP with properties that are potentially advantageous for life science and therapeutic applications.

Cell-penetrating peptides (CPPs) overcome the impermeability of the plasma membrane, allowing drugs to be delivered to cells and tissues^1^,^2^,^3^. They are generally 10 to 30 amino acid (aa) residues in length, and either arginine-rich, amphipathic and lysine-rich, or hydrophobic^4^. They are not cell-type specific, using multiple pathways to traverse the plasma membrane^5^. Several widely studied CPPs were derived or reconstructed from viral proteins, including Tat peptide (TATp) and oligoarginine^6^,^7^, MPG peptides and Pep1^8^,^9^, and VP22^10^. Since the discovery of TATp in 1988^6^, CPPs have been shown to be capable of delivering a wide variety of different cargo types to cells in culture and within living animals^1^,^2^,^3^.

The 53 aa residue X-protein is one of just seven proteins encoded by the hepatitis B virus (HBV)^11^, the smallest known DNA virus which chronically infects 400 million people worldwide, one million of whom die annually from HBV-related liver disease^11^,^12^. Similarities can be drawn between the X-protein and the Tat protein of the human immunodeficiency virus (HIV). Both proteins significantly increase the level of transcription of their respective viral RNAs, and they are both small proteins that contribute to the development of virally-induced cancers, namely Karposi's sarcoma and hepatocellular carcinoma, respectively. However, while Tat is cell-permeable^6^, the wild-type X-protein is not^13^, which might suggest that the X-protein lacks a cell-penetrating domain.

We set out to identify functional domains within the X-protein by screening short peptides encompassing the 154 aa residue X-protein for activity. Quite serendipitously, we discovered an X-protein peptide that was inherently cell-permeable. Here we report on the unique functional properties of this peptide, and its ability to deliver therapeutic cargoes.

## Results

### The N-terminal region of the X-protein harbours a cell-penetrating peptide that penetrates adherent cells, but not nonadherent resting blood cells

**Figure 1:**
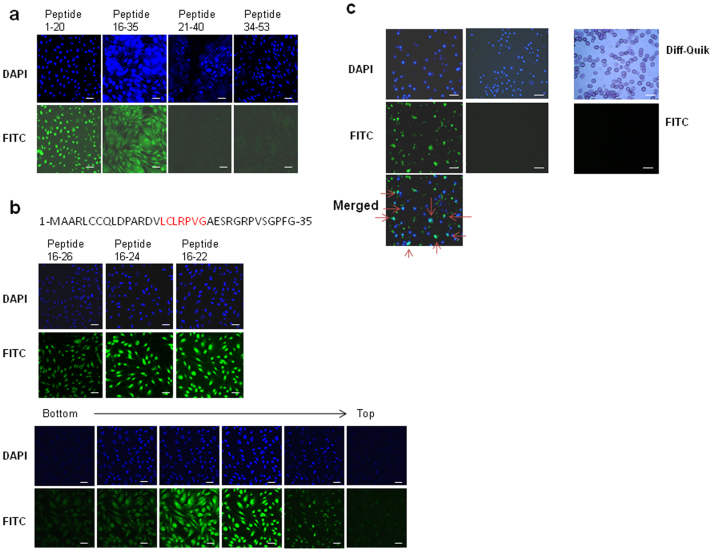
Cell-permeability of X-protein peptides. (a) X-protein peptides aa 1–20 and 16–35 are cell-permeable. Four FITC-labelled peptides encompassing aa 1–20, 16–35, 21–40 and 34–53 from the N-terminal region of the X-protein were incubated at 10 μM with HepG2 cells and their uptake by the cells recorded by confocal microscopy. Cell nuclei were stained blue with DAPI. (b) The sequence of the first 35 N-terminal residues of the HBV X-protein are shown with the sequence of Xentry (LCLRPVG; residues 16–22) highlighted in red. Short FITC-labelled peptides (10 μM) aa 16–26, 16–24 and 16–22 (Xentry) derived from peptide aa 16–35 also permeate HepG2 cells (upper panel). Confocal slicing of HepG2 cells reveals that peptide aa 16–22 (Xentry) is taken up into the cytoplasm and nucleus (lower panel). Cell nuclei were stained blue with DAPI. (c) FITC-labelled Xentry (10 μM) permeates adherent blood monocytes (left panel; arrows denote cells that have taken up Xentry), but not non-adherent blood lymphocytes (middle panel) or erythrocytes (right panel; erythrocytes stained with Diff-Quik). Cell nuclei were stained blue with DAPI. Scale bar, 50 μm.

The wild-type X-protein is not cell-permeable^13^, hence, it was a surprise to discover that two overlapping FITC-labelled peptides spanning aa residues 1–20 and 16–35 of the X-protein were able to permeate HepG2 cells (Fig. 1a). The peptides were taken up by cells within minutes, localizing to both the cytoplasm and nucleus. In contrast, C-terminal peptides 21–40 and 34–53 were not cell-permeable. The sequence LCLRP (aa 16–20) was common to both cell-permeable peptides, and accordingly peptides encompassing residues 16–26, 16–24, and 16–22 were each cell-permeable, as viewed by confocal microscopy (Fig. 1b). The 7 amino acid residue peptide LCLRPVG (residues 16–22), designated Xentry (Fig. 1b), was capable of permeating a wide variety of immortalized and cancerous cell lines, including HepG2 (liver), C32 (melanoma), DU145 (prostate), H441 (lung), BT474 (breast), Cos (kidney), and Rin-m5F (pancreatic β-cell) cell lines (data not shown). In stark contrast, Xentry was incapable of permeating non-adherent human peripheral blood lymphocytes and erythrocytes (Fig. 1c), K562 erythroleukemia cells, and mouse TK-1 T cells (Supplementary Fig. 1). However, it was taken up by peripheral blood monocytes attached to plastic (Fig. 1c), and by Mn^++^-activated adherent TK-1 T cells that had been bound via α4β7 to MAdCAM-1-coated plates (Supplementary Fig. 1).

### Xentry penetrates living HepG2 cells

**Figure 2:**
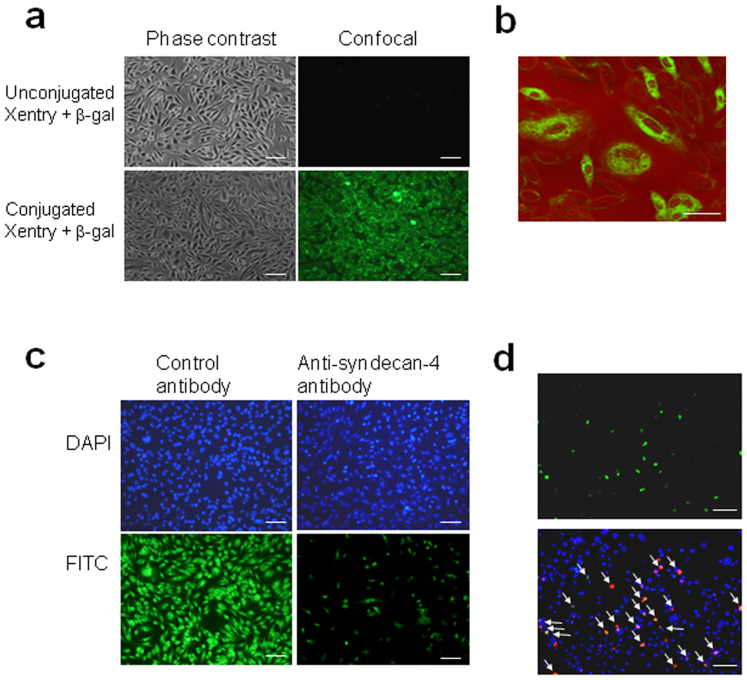
Evidence that a Xentry-β-galactosidase conjugate is internalized by living HepG2 cells, and that Xentry enters cells via a syndecan pathway. (a) Visualization of the internalization of an unlabelled Xentry-β-galactosidase conjugate, and unconjugated β-galactosidase, after a 10 min incubation with live cells preloaded with the green fluorescent substrate C_12_FDG. (b) Confocal microscopy reveals the Xentry-β-galactosidase conjugate is taken up into the cytoplasm, but excluded from the nucleus. (c) Uptake of FITC-labelled Xentry (10 μM) by HepG2 cells is blocked by treatment of cells with 1 μg/ml of anti-syndecan-4 antibody, whereas 1 μg/ml of a control antibody against TRAIL has no effect. (d) Syndecan-deficient K562 cells transfected to express syndecan-4 show increased uptake of TAMRA-labelled (red) Xentry (20 μM). Syndecan-4-expressing transfectants were detected with a rabbit polyclonal anti-syndecan 4 antibody, followed by a FITC-labelled (green) donkey anti-rabbit antibody. Cell nuclei were stained blue with DAPI. Arrows mark syndecan 4-expressing transfectants that have taken up TAMRA-labelled Xentry. Scale bar, 50 μm.

Fixation artifacts resulting from cell processing have historically led to misinterpretation of the internalization of CPPs^14^. Hence a living cell assay based on the intracellular loading of C_12_ fluorescein di-β-D-galactopyranoside (C_12_FDG), a cell-permeable substrate for β-galactosidase, was undertaken to confirm that Xentry could deliver β-galactosidase to cells. In this assay, a fluorescent signal is generated by internalized β-galactosidase in living unfixed cells, and not by membrane-bound or extracellular enzyme. Xentry was modified by incorporation of a stretch of glutamine residues and conjugated to recombinant bacterial β-galactosidase via transglutamination. β-galactosidase conjugated to Xentry, but not unconjugated β-galactosidase, was taken up by live HepG2 cells that had been preloaded with C_12_FDG, causing the cells to fluoresce bright green (Fig. 2a). Confocal microscopy revealed that the conjugate was confined to the cell cytoplasm, unlike the free Xentry peptide which reached the cell nucleus, indicating that the nature of the cargo affects the subcellular distribution of the conjugate (Fig. 2b).

### Cell uptake of Xentry is an energy-dependent process involving heparan sulfate proteoglycans and clathrin

The cellular requirements for internalization of Xentry by HepG2 cells were assessed to obtain insight into the mechanism of cell entry. Xentry was internalized by HepG2 cells at 37°C, but not at 4°C, indicating that its uptake involves an energy-dependent endocytic process, and accordingly uptake was blocked by pretreatment of cells with the combination of azide and deoxyglucose (Supplementary Fig. 2). The importance of glycosaminoglycans to internalization was demonstrated by the finding that cell uptake of Xentry was blocked with heparin (Supplementary Fig. 2). This result is in accord with the finding that Xentry cannot penetrate K562 cells (Supplementary Fig. 1), which are deficient in cell-surface heparan sulfate proteoglycans (HSPGs) except for minor amounts of endogenous betaglycan^15^,^16^. Uptake was also blocked with chlorpromazine that inhibits the clathrin pathway of endocytosis (Supplementary Fig. 2). Cytochalasin D, an actin depolymerizing agent, and the chemical filipin which blocks caveolae-mediated uptake and inhibits macropinocytosis were without effect (data not shown). The latter is in accord with the fact that HepG2 cells do not express caveolin^17^, providing evidence that caveolae are not required.

### Cell uptake of Xentry is mediated by syndecans

The above results suggested that Xentry enters cells via a ubiquitous HSPG that is internalized by the clathrin pathway, and upregulated *de novo* on activated blood cells. The universally expressed syndecan isoform syndecan-4 (ryudocan, amphiglycan) exhibits these properties. It has been reported to bind and transport the cationic CPPs penetratin, octaarginine and TATp into the cells via its heparan sulfate chains^16^. It seemed a likely candidate receptor for Xentry. In accord, uptake of Xentry into HepG2 cells was blocked by a polyclonal anti-syndecan-4 antibody (Fig. 2c). Further, Xentry was exclusively taken up by syndecan-deficient K562 cells transfected to express syndecan-4 (Fig. 2d).

### Xentry delivers therapeutic antibodies and siRNAs to cells

**Figure 3:**
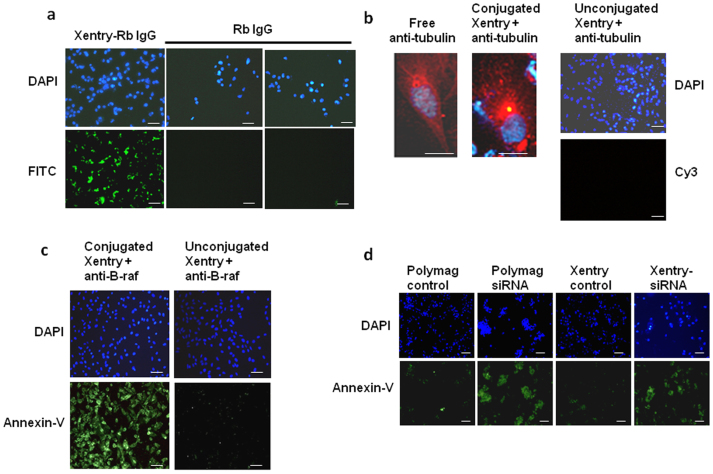
Xentry-mediated delivery of antibody and siRNA cargoes into cells. (a) Uptake of Xentry-conjugated and unconjugated FITC-labelled rabbit IgG by HepG2 cells. Cell nuclei were stained blue with DAPI. (b) Representative cell showing staining of tubulin by a Cy3-labelled anti-β-tubulin antibody delivered to HepG2 cells by Xentry. HepG2 cells were incubated with the Xentry- anti-β-tubulin antibody conjugate for 1 h, washed, then incubated and photographed 24 h later. For comparison, a representative fixed and permeabilized cell is shown which has been stained by the free anti-β-tubulin antibody. The unconjugated anti-β-tubulin antibody could not penetrate and stain non-permeabilized HepG2 cells. (c) The abilities of a Xentry-conjugated and unconjugated anti-B-raf antibody to induce the apoptosis of WM-266-4 melanoma cells, as evidenced by staining of cells with annexin-V fluos (green). (d) A Xentry-KALA fusion peptide delivers an siRNA directed against B-raf transcripts into WM-266-4 melanoma cells, causing cell apoptosis as evidenced by staining of cells with annexin-V fluos (green). For comparison, the anti-B-raf siRNA was transfected into cells using the PolyMag agent. The siRNA was omitted from controls. Scale bar, 50 μm.

A key question was whether Xentry would be able to deliver a large therapeutic cargo such as an antibody to cells. FITC-labelled purified IgG from the serum of an unimmunized rabbit (Fig. 3a), and a Cy3-labelled anti-β-tubulin antibody (Fig. 3b) conjugated to Xentry were taken up into the cytoplasm of HepG2 cells, but unlike the free peptide, were restricted from entering the nucleus. The antibody complexes were not toxic to cells (data not shown). The Cy3-labelled anti-β-tubulin antibody gave a similar profile of staining to that obtained by staining fixed and permeabilized cells with the free antibody, where staining of microtubules could be discerned (Fig. 3b). Xentry was conjugated to an anti-B-raf antibody in order to demonstrate that it could deliver a therapeutic antibody in a biologically active form to melanoma cells. Addition of conjugated, but not unconjugated anti-B-raf antibody, caused WM-266-4 melanoma cells whose survival depends upon B-raf^18^, to undergo apoptosis (Fig. 3c). Xentry was fused to a KALA peptide, which is able to spontaneously bind RNA^19^. Xentry-KALA peptide-mediated delivery of a double-stranded siRNA against B-raf also caused the melanoma cells to undergo apoptosis, to a similar extent as that obtained by polyMag-mediated transfection of the B-raf siRNA (Fig. 3d).

### Biodistribution of Xentry intravenously injected into mice

**Figure 4:**
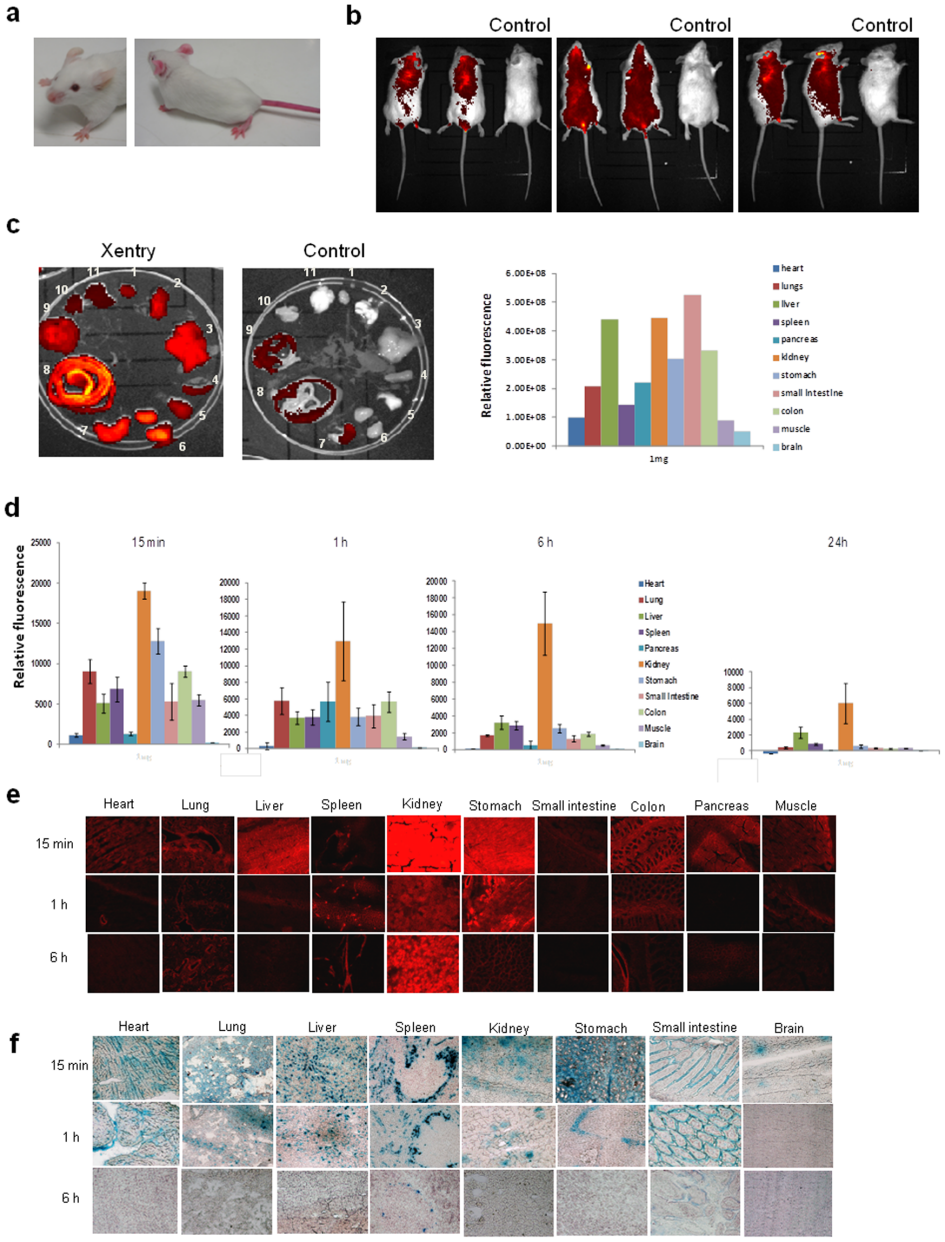
Biodistribution of Xentry, and a Xentry-β-galactosidase conjugate intravenously injected into mice. (a) The hairless extremities of mice (right photograph) intravenously injected with 5 mg of Cy7 (red)-labelled Xentry assumed a bright red colour under natural light, compared to those of uninjected mice (left photograph). Mice were intravenously injected with 1 mg of Cy7-labelled Xentry, and the fluorescence of the whole bodies (b) and major organs (c) were recorded using the Xenogen-IVIS® Kinetic-In Vivo Imaging System, and compared with untreated control littermates. Dorsal, ventral and side views of representative mice are shown. The relative levels of fluorescence of the organs were plotted. The organs are in numerical order starting with heart (1) and ending with brain (11) as in the vertical listing. Mice were intravenously injected with 1 mg of TAMRA-labelled Xentry and the fluorescence of organ homogenates (d) and sections (e) was measured 15 min, 1, 6, and 24 h later. The relative levels of fluorescence of the organs were plotted as the mean ± SD (n = 3). (f) Mice were intravenously injected with 1 mg of β-galactosidase conjugated to Xentry, and β-galactosidase activity in sections of organs collected at 15 min, 1 and 6 h was visualized by staining tissue sections with the β-galactosidase substrate X-gal (blue).

Xentry would need to be stable in serum to be of value for drug delivery, hence it was synthesized as a D-isomer to increase its resistance to proteases. The D-isomeric form was cell-permeable, displaying increased uptake into HepG2 cells at increasing concentrations (Supplementary Fig. 3a). It was stable in serum for at least 4 h, whereas the L-isomer was rendered inactive within 1 h (Supplementary Fig. 3b). A Cy7-labelled D-isomeric form of Xentry was injected into mice via the tail vein to examine its biodistribution and pharmacokinetic properties. The extremities of mice including the tail, feet, nose and ears assumed a bright red colour under natural light due to retention of the red-coloured peptide (Fig. 4a). Both the urine and faeces of mice were coloured bright red. The whole bodies (Fig. 4b) and organs (Fig. 4c) of mice intravenously injected with 1 mg of Cy7-labelled Xentry were analyzed for retention of Xentry at 15 min, 1 h (Fig. 4b, c), 6 h, and 24 h using the Xenogen-IVIS® Kinetic-In Vivo Imaging System (Supplementary Fig. 4). Xentry injected at doses of 0.1 and 1 mg was rapidly taken up by all major tissues examined within 15 min, including the heart, lungs, liver, spleen, pancreas, kidneys, stomach, small intestine, colon, muscle, and brain (Fig. 4b, c; Supplementary Fig. 4). It was retained to some extent at 24 h by all organs at the higher dose, but had been almost completely excreted by 6 h at the lower dose (Supplementary Fig. 4).

Mice were intravenously injected with 0.1, 1 and 5 mg of TAMRA-labelled Xentry and their major organs were analyzed for retention of Xentry at 15 min, 1 h, 6 h, and 24 h by recording the fluorescence in organ homogenates and sections (Fig. 4d, e; Supplementary Fig. 5). Analysis of organ homogenates revealed that Xentry was rapidly taken up within 15 min at each of the 3 doses by all major tissues examined including the lungs, liver, spleen, kidneys, stomach, small intestine, colon, muscle, but less well by heart, pancreas, brain (Fig. 4d). As described above, the levels of Xentry gradually declined such that by 24 h it was present at only low levels in most organs, except for liver and kidneys. Retention of higher levels of peptide in the liver and kidneys is expected as intravenously injected small peptides are cleared via either renal or hepatobiliary excretion or both, depending upon their hydrophilicity or hydrophobicity^20^. Tissue sections of organs revealed the presence of Xentry in most organs (Fig. 4e). Xentry appeared to be concentrated within particular regions of certain tissues, such as the epithelia lining the colon, stomach and bronchial airways. Cytospins of whole blood made at 15 min, 1 h, 6 h and 24 h confirmed that Xentry was not taken up and sequestered by circulating blood cells (Supplementary Fig. 6).

### Biodistribution of a Xentry-β-galactosidase conjugate intravenously injected into mice

Mice were intravenously injected with 1 mg of a Xentry-β-galactosidase conjugate in order to compare the biodistribution of free Xentry peptide with that of Xentry conjugated to a large protein cargo (Fig. 4f). A control mouse was injected with 1 mg of unconjugated β-galactosidase. Organs were recovered at 15 min, 1 h, 6 h, and 24 h and sections stained with the β-galactosidase substrate X-gal. At 15 min following injection of the conjugate most of the organs stained blue with X-gal, including the lungs, liver, spleen, kidneys, stomach, small intestine, colon, muscle, and brain (Fig. 4f), whereas the pancreas, colon, and muscle were poorly stained (data not shown). All the stained organs retained the bioactive conjugate at the 1 h time point, except for the brain, albeit the intensity of staining was diminished. At 6 h only the spleen and small intestine retained some β-galactosidase activity, and by 24 h activity was completely lost (data not shown). The staining pattern of certain organs was quite distinctive. The conjugate appeared to be concentrated in the enterocytes of the small intestine, epithelium of the stomach, and in the marginal zone or marginal sinus of the spleen with exclusion from lymphocyte-containing follicles as expected (Fig. 4f). Organs from mice injected with unconjugated β-galactosidase did not stain with X-gal (data not shown).

## Discussion

Xentry represents a new class of CPP in that it is very short, has only a single charged arginine residue, and bears no resemblance to any previously described CPP. Arginine is the main contributor of charge for Tat, penetratin and pVEC, whereas lysine contributes charge to transportan and MAP^1^,^2^,^3^,^4^. Unlike other CPPs Xentry enters cells exclusively via an energy-dependent endocytic process involving HSPG-decorated syndecan-4, which explains its inability to permeate unactivated non-adherent blood cells. It appears to bind the glycan side-chains of syndecan-4 as its uptake into cells is blocked with heparin. In accord, syndecan is not expressed on circulating and peripheral B cells, but its expression is induced upon B cell differentiation into plasma cells^21^. Syndecan is hypoglycosylated on resting T cells and upregulated and glycosylated on cell activation^22^. Other CPPs can permeate blood cells as they exploit multiple pathways of cell uptake including the caveolae pathway, macropinocytosis, and direct translocation across the plasma membrane^5^,^23^. Cell uptake of Xentry is therefore likely to be highly controllable, which will be important for potential tumour-activatable forms or other tissue-targeted forms where tissue-specific uptake has to be tightly controlled for the delivery of highly toxic therapeutics. The inability of Xentry to penetrate resting blood cells promises a therapeutic advantage, as Xentry is not sequestered and diluted when administered intravenously, thereby increasing tropism for tissues and tissue-specific targeting.

The data indicate that the binding of Xentry to heparan sulphate moieties on syndecan-4 triggers the uptake of Xentry via the clathrin pathway. HSPGs and syndecans are known for their ability to internalize physiological extracellular ligands, viruses, bacteria and basic peptides^24^. Syndecans are reported to internalize cargoes via clathrin-independent pathways^24^, but this is not always the case as exemplified by the ability of R-Spondin to induce syndecan-4-dependent, clathrin-mediated, endocytosis^25^.

The cell-permeability of the Tat protein is thought to be important in HIV infection, as the Tat protein is capable of leaving an infected cell and entering other cells and inducing viral gene transcription, immunosuppression, or cell death^26^,^27^. The native X-protein is not cell-permeable, indicating that the Xentry sequence must be masked in some way. It remains to be seen whether any of the many natural splice variants of the X-protein are cell-permeable, and also contribute to viral gene transcription and tumorigenesis^6^.

Xentry should have application in delivering drugs to tissues in the treatment of disease, since many different cell types express syndecans, in particular epithelia which express high levels of syndecans. It could be administered locally to target a particular tissue (eg the skin, or bronchial epithelium), or fused to a growing list of tissue-specific homing peptides to aid targeting^28^,^29^. A tumour-activatable form of Xentry would be particularly useful in the treatment of cancer, as elegantly devised for the polyarginine class of CPPs^30^. Despite its small size, Xentry is capable of delivering large proteins, antibodies, and siRNAs into cells in a biologically active form. In the first human clinical trial involving a CPP, a conjugate of a protein kinase C δ inhibitor and TATp was shown to safely improve recovery following myocardial infarction^31^. In terms of safety, Xentry caused no signs of acute toxicity when delivered at very high concentrations in a single injection into the circulation, but its safety in terms of prolonged continuous delivery remains to be determined. Xentry is a new class of CPP and may offer advantages in situations where other CPPs are found to be unsuitable.

## Methods

### Assay to test for cell permeability of peptides

HepG2 cells (ATCC, Manassas, VA) were seeded into 8-well chamber slides at 1 × 10^5^ cells per well in MEM media (Gibco, Life Technologies New Zealand Ltd) containing 10% FCS and PSG. They were cultured overnight at 37°C in a 5% CO_2_ atmosphere, and washed thrice with serum-free MEM media. L-isomeric peptides were diluted in 500 μl of MEM media without FCS, whereas D-isomeric peptides were diluted in the same media containing FCS, and each added to cells at a final concentration of 10 μM, or as indicated. The cells were incubated for 3 h at 37°C in a 5% CO_2_ atmosphere, washed with PBS, fixed with 4% formaldehyde for 30 min, and washed thrice with PBS. A drop of Prolong Gold anti-fade reagent with DAPI (Invitrogen, Life Technologies New Zealand Ltd) was added, and the cells mounted and examined with a Nikon E600 fluorescence microscope or a Leica TCS-SP2 confocal microscope.

### Visualization of uptake of Xentry-β-galactosidase conjugate within live HepG2 cells

HepG2 cells were seeded at 1 × 10^5^ cells per well into 8-well chamber slides, and cultured overnight at 37°C in a 5% CO_2_ atmosphere. They were washed thrice with serum-free MEM media, and 500 μl of serum-free MEM added to the wells. A Xentry peptide (biotin-LCLRPVGGGRRRQQQQQQRRR; Peptide 2.0 Inc., Chantilly, VA) where LCLRP was fused to a polyglutamine stretch was conjugated to recombinant β-galactosidase. A 30 μl solution containing conjugation buffer (5 mM CaCl_2, _1 mM DTT, 50 mM Tris-HCl), 0.84 μg of Xentry peptide, 16.5 μg of β-galactosidase (5 U; Sigma, MO), and 4.8 μg of transglutaminase (Ajinomoto, Itasca, IL) was incubated for 1 h at 37°C. The resulting conjugate was added to the cells, which were incubated for 3 h at 37°C. The cells were washed thrice with PBS, and the β-galactosidase substrate 5-dodecanoylaminofluorescein di-β-D-galactopyranoside (Imagene C_12_FDG; Invitrogen Life Technologies New Zealand Ltd) added, followed by incubation for 10 min at 37°C. The cells were washed, and examined using a Nikon E600 fluorescence microscope and photographed.

### Test for Xentry-mediated delivery of rabbit IgG and anti-B-raf antibodies

HepG2 cells were seeded at 1 × 10^5^ cells per well into 8-well chamber slides, and cultured overnight at 37°C in an atmosphere of 5% CO_2_. They were washed thrice with serum-free MEM media and resuspended in 500 μl of serum-free MEM. A 30 μl solution containing conjugation buffer, 0.85 μg of the Xentry peptide biotin-LCLRPVGGGRRRQQQQQQRRR, 9 μg of a FITC-labelled rabbit IgG (Sigma, MO) or 1 μg of a rabbit polyclonal anti-B-raf antibody (Sigma, MO), and 4.8 μg of transglutaminase was incubated for 1 h at 37°C. The resulting conjugates were added to the cells, which were incubated for 3 h at 37°C and washed thrice with PBS. Cells incubated with the Xentry-rabbit IgG conjugate were fixed with 4% formaldehyde, washed thrice with PBS and DAPI added for visualization of cell nuclei. Cells incubated with the Xentry-anti-B-raf antibody conjugate were stained with annexin-V Fluos (Roche Diagnostics NZ Ltd., Auckland) to detect cell apoptosis. The cells were examined using a Nikon E600 fluorescence microscope, and photographed.

### Testing the ability of a Xentry-KALA fusion peptide to deliver an anti-B-raf siRNA into cells

Wm233-4 melanoma cells were seeded at 1 × 10^5^ cells per well into 8-well chamber slides, and cultured overnight at 37°C in an atmosphere of 5% CO_2. _They were washed thrice with serum-free MEM media and resuspended in 500 μl of the same media. A 600 μl solution was prepared containing 30 nM of a Tex615-labelled siRNA against B-raf (sense 5′-XAGAAUUGGAUCUGGAUCAUTTY-3′; antisense 5′-AUGAUCCAGAUCCAAUUCUTT-3′; X = C12 amino, Y = Tex615) (Integrated DNA Technologies, Iowa) and 10 μM of a Xentry-KALA fusion peptide (biotin-LCLRPVGGGWEAKLAKALAKALAKHLAKALAKALKACEA; Peptide 2.0 Inc., Chantilly, VA). This resulting solution was incubated at room temperature for 20 min to allow the siRNA to bind the Xentry-KALA fusion peptide. The Xentry-KALA-siRNA complex was added to the cells, followed by incubation at 37°C for 72 h, with a media change at 24 and 48 h. As a control, an equivalent amount of siRNA was transfected into cells using the commercial transfection reagent Polymag (OZ Biosciences, Marseille, France). After 3 h the media was changed to MEM containing 10% FCS and PSG, and the cells were incubated overnight at 37°C in a 5% CO_2_ atmosphere. The media was changed at 24 and 48 h. Controls included reactions in which the siRNA was omitted. Cells were stained with annexin-V Fluos to detect apoptotic cells by fluorescence microscopy, and photographed. Note: The siRNA was not active when directly conjugated to Xentry.

### Biodistribution of TAMRA-labelled Xentry in mice

All experiments in mice were performed in accordance with relevant institutional guidelines and regulations, and were subject to a protocol approved by the Animal Ethics Committee, the University of Auckland. Different amounts (0.1 mg, 1 mg and 5 mg) of a TAMRA-labelled D-isomeric form of Xentry (TAMRA-lclrpvg; Peptide 2.0 Inc., Chantilly, VA) were dissolved in 50 μl of PBS. The peptide was administered intravenously via the tail vein into groups of Balb/c mice (3 mice per time-point), which were euthanized at 15 min, 1, 6 and 24 h. Three mice were injected with 50 μl of PBS to serve as controls. Cardiac blood was collected into an EDTA tube and cytospun onto slides, and viewed by fluorescence microscopy to determine uptake of the peptide by blood cells. The major organs of each mouse was harvested and dissected in half to be either homogenized or placed in a cryotube with isopentane, frozen and sectioned. For homogenization, the organs were weighed and then homogenized in 500 μl of PBS in an Omni bead-ruptor homogenizer (OMNI International, Kennesaw, GA). Equal volumes of homogenate and 8 M guanidinium chloride were mixed, and then centrifuged at 12,000 g for 5 min, and fluorescence of the supernatant measured in a fluorescence plate reader.

### Biodistribution of Cy7-labelled Xentry in mice recorded using a IVIS Kinetic machine

Different amounts (0.1 mg, 1 mg) of a Cy7-labelled D-isomeric form of Xentry (Cy7-lclrpvg, Auspep, Tullamarine VIC) were dissolved in 50 μl of PBS. The peptide was administered intravenously via the tail vein into groups of Balb/c mice (3 mice per time-point), which were euthanized at 15 min, 1, 6 and 24 h. Four mice, one at each time-point, were injected with 50 μl of PBS to serve as controls. The mice were then placed into the Xenogen-IVIS® Kinetic-In Vivo Imaging System (Caliper Life Sciences, Hopkinton, MA) and fluorescence images taken of dorsal, ventral and lateral views. Fluorescence images were also taken of the major organs of each mouse. Images were analyzed using Living Image (Caliper Life Sciences, Hopkinton, MA) software.

### Biodistribution of a Xentry-β-galactosidase conjugate in mice

One milligram of recombinant β-galactosidase (Medicago AB, Uppsala, Sweden) was cross-linked to 0.2 mg of a FITC-labelled Xentry peptide fused to a polyglutamine stretch (FITC-lclrpvggrrQQQQQQrr; Peptide 2.0 Inc., Chantilly, VA) with 4.8 μg of transglutaminase in 40 μl of transglutaminase buffer for 1 h at 37°C. The Xentry-β-galactosidase conjugate was intravenously injected via the tail vein into groups of Balb/c mice (3 mice per time-point), which were euthanized at 15 min, 1, 6 and 24 h. Control mice (one mouse per time point) were intravenously injected with 1 mg of unconjugated β-galactosidase. The heart, lungs, liver, spleen, pancreas, kidneys, stomach, small intestine, colon, muscle and brain of each mouse were collected, and frozen at −80°C. Frozen organ sections were fixed in 0.25% glutaraldehyde for 15 min, washed thrice with PBS and stained with 1 mg/ml of X-gal (Sigma, MO) for 3 h at 37°C. The sections were washed thrice with PBS, examined by light microscopy and photographed.

### Anti-syndecan 4 antibody blockade of Xentry uptake by HepG2 cells

HepG2 cells were seeded at 1 × 10^5^ cells per well into 8-well chamber slides, and cultured overnight at 37°C in a 5% CO_2_ atmosphere. The media was replaced with 500 μl of MEM media containing 10% FCS and either 1 μg/ml of **a** goat anti-human syndecan-4 antibody (R&D Systems, Minneapolis, MN), or 1 μg/ml of the control goat anti-human TNF-related apoptosis-inducing ligand (TRAIL) antibody (Santa Cruz Biotechnology Inc., Santa Cruz, CA). The cells were then incubated at 37°C and 5% CO_2 _for 30 min. FITC-labelled Xentry (FITC-lclrpvg) was added at a final concentration of 10 μM and the cells incubated for 3 h at 37°C in a 5% CO_2_ atmosphere. The cells were washed thrice with PBS, fixed with 4% formaldehyde for 15 min, washed with PBS, and DAPI added for visualization of cell nuclei. They were examined using a Nikon E600 fluorescence microscope, and photographed.

### Testing uptake of Xentry by K562 cells engineered to express syndecan-4

K562 cells in RPMI media containing 10% FCS were seeded at 1 × 10^5^ cells per well into 6-well low attachment tissue culture plates (BD Biosciences, Franklin Lakes, NJ), and cultured overnight at 37°C in a 5% CO_2_ atmosphere_. _The media was removed and replaced with RPMI without serum. Four micrograms of the syndecan-4 plasmid pCMV-hygro-SDC4 and 4 μl of polymag transfection reagent (OZ Biosciences, Marseille, France) were mixed in 100 μl of Opti-MEM media. The mixture was incubated for 20 min at room temperature, added to the cells, and the tissue culture plate placed onto a magnetic plate for 20 min. The media was removed and replaced with RPMI media containing 10% FCS, followed by incubation for 48 h at 37°C and 5% CO_2, _with a media change after 24 h. TAMRA-labelled Xentry (TAMRA-lclrpvg) was added to the transfectants 48 h following transfection to a final concentration of 20 μM, and the cells were incubated for 3 h. The cells were removed from the plate, washed thrice with PBS, cytospun onto a slide, and fixed with 4% formaldehyde for 15 min. They were washed with PBS and DAPI added for visualization of cell nuclei. Syndecan-4-expressing transfectants were detected with a rabbit polyclonal anti-syndecan 4 antibody (IBL-Japan), followed by a FITC-labelled donkey anti-rabbit antibody. The cells were then examined using a Nikon E600 fluorescent microscope, and photographed.

## Author Contributions

G.W.K. conceived and directed the research. K.M. and Y.Y. conducted the *in vitro* assays, and K.M. and X.S. conducted the animal work. S.W. helped with the Kinetic analysis. G.W.K., K.M., Y.Y. and X.S. analyzed the data. G.W.K. and K.M. wrote the manuscript. All authors commented on the manuscript.

## Supplementary Material

Supplementary Information Supplementary figures
