# Supplementary material for: Xentry, a new class of cell-penetrating peptide uniquely equipped for delivery of drugs
Source: Sci Rep. 2013 Apr 16;3:1661. doi: 10.1038/srep01661 (PMC3627194; doi:10.1038/srep01661)
Supplement: Supplementary Information — Supplementary figures [file srep01661-s1.pdf]

Xentry, a new class of cell-penetrating peptide uniquely equipped for delivery of drugs

Kristopher Montrose, Yi Yang, Xueying Sun, Siouxsie Wiles, and Geoffrey W. Krissansen

[1]Department of Molecular Medicine & Pathology, Faculty of Medical and Health Sciences, University of Auckland, Auckland 1005, New Zealand.

Supplementary Figure 1

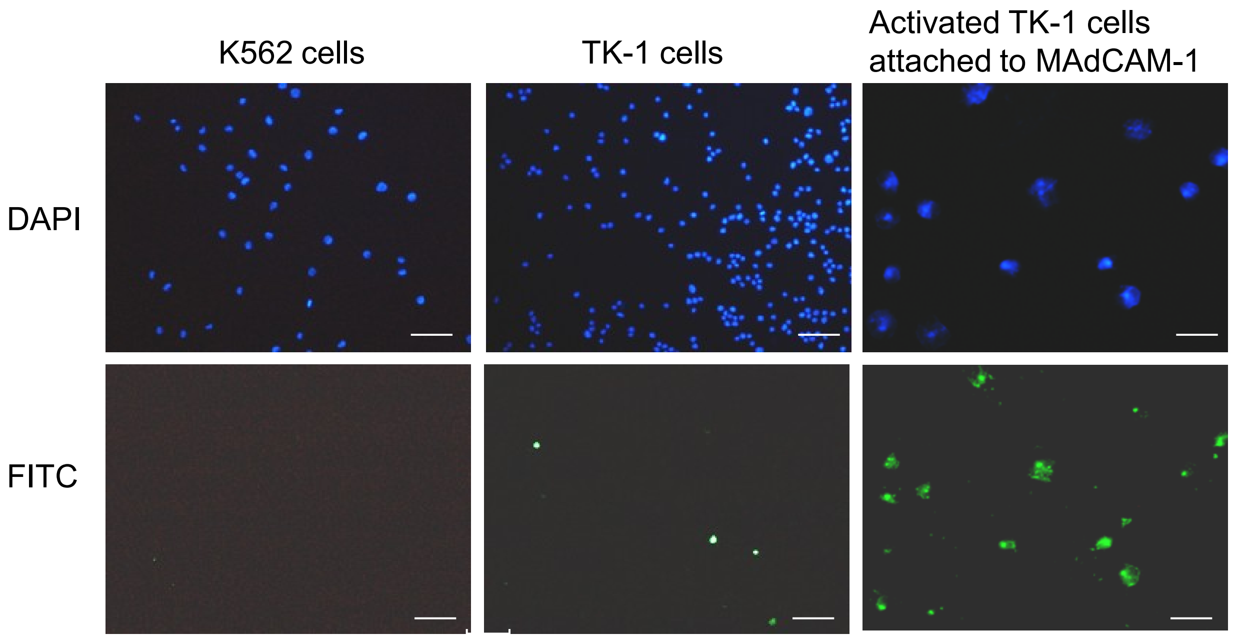

Supplementary Figure 1. Xentry does not permeate non-adherent blood cells. FITC-labelled Xentry (LCLRPVG; aa 16-22) was incubated with non-adherent K562 erythroleukemia cells and mouse TK-1 T cells and with Mn^{++}-activated TK-1 cells attached to MAdCAM-1-coated plates. Cell uptake was recorded by fluorescence microscopy. Cell nuclei were stained blue with DAPI. Plates were coated with 1 µg/ml of MAdCAM-1 overnight and activated TK-1 cells were allowed to bind to the plates for 45 mins before incubation with FITC-labelled Xentry for 3 h. Scale bar represents 50 µm.

Supplementary Figure 2

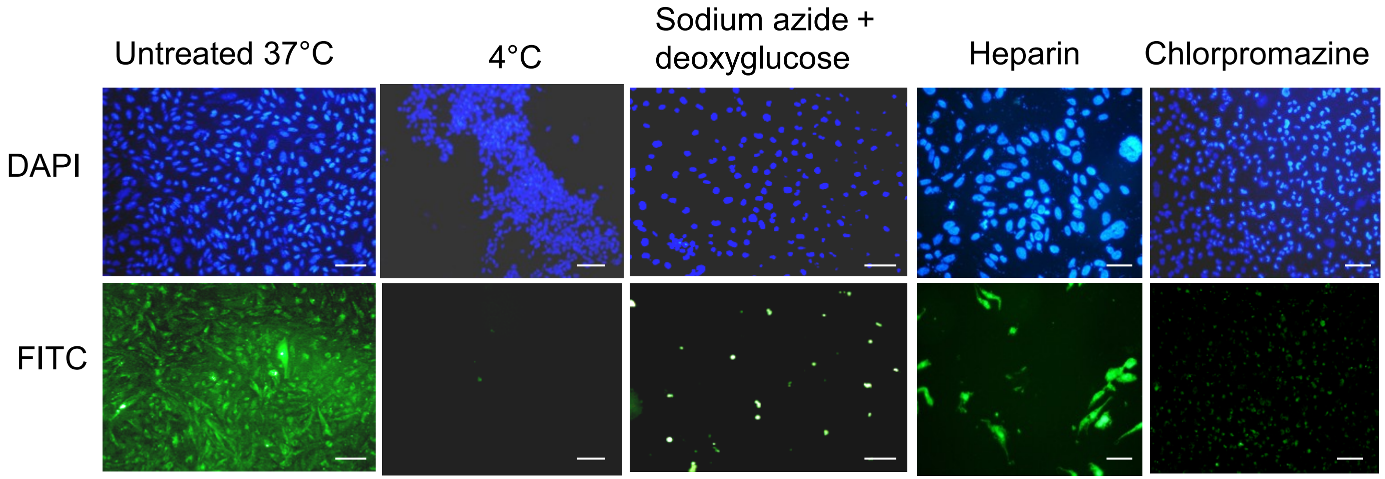

Supplementary Figure 2. Cellular requirements for internalization of Xentry by HepG2 cells. **(a)** Incubation of FITC-labelled Xentry with HepG2 cells for 3 h at 4°C, or in the presence of 10 mM sodium azide and 6 mM 2-deoxy-D-glucose, 2 µg/ml heparin, or 10 µg/ml of chlorpromazine, as indicated, inhibited peptide uptake. Cell nuclei were stained blue with DAPI. Scale bar, 50 µm.

Supplementary Figure 3

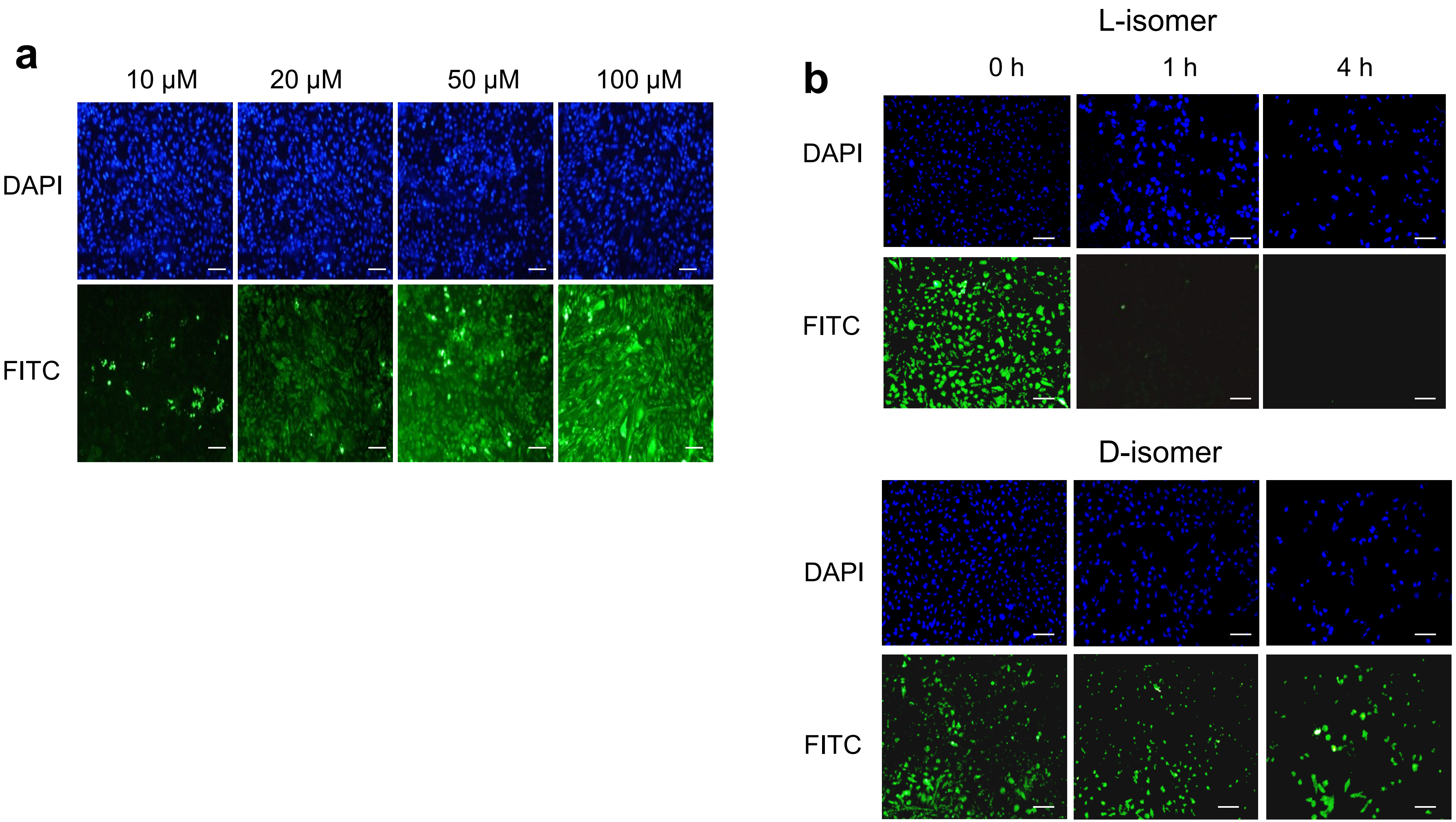

Supplementary Figure 3. The D-isomeric form of Xentry aa 16-22 is cell permeable, and is stable in serum for at least 4 h. **(a)** FITC-labelled D-isomeric Xentry was incubated with HepG2 cells at concentrations of 10, 20, 50 and 100 μm for 3 h. Uptake was recorded by fluorescence microscopy. Cell nuclei were stained with DAPI. (**b**) FITC-labelled L-isomeric and D-isomeric forms of Xentry were incubated in serum for 0, 1, and 4 h before incubation with HepG2 cells. Cell nuclei were stained with DAPI. Scale bar, 50 μm.

Supplementary Figure 4

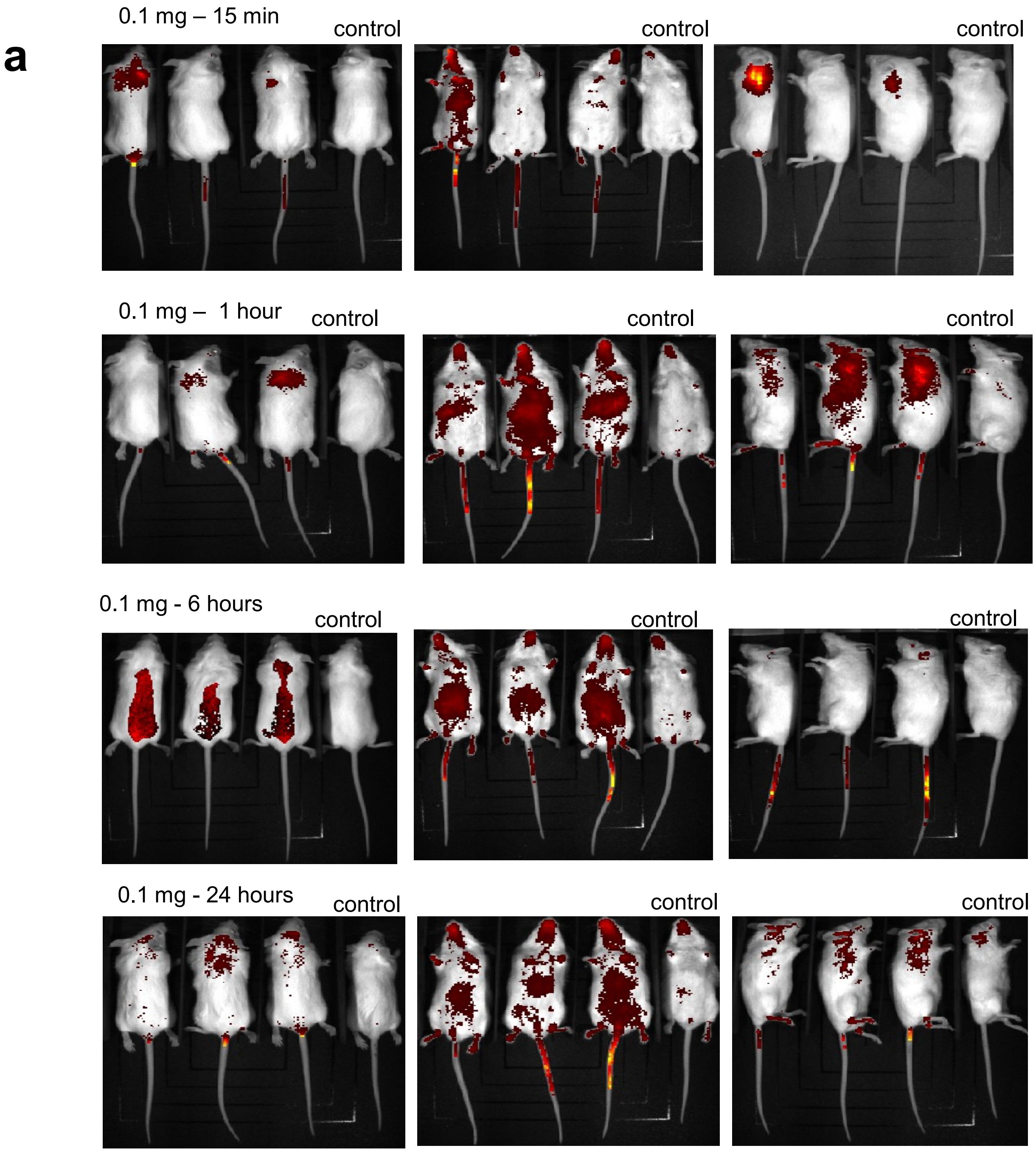

Supplementary Figure 4 (cont)

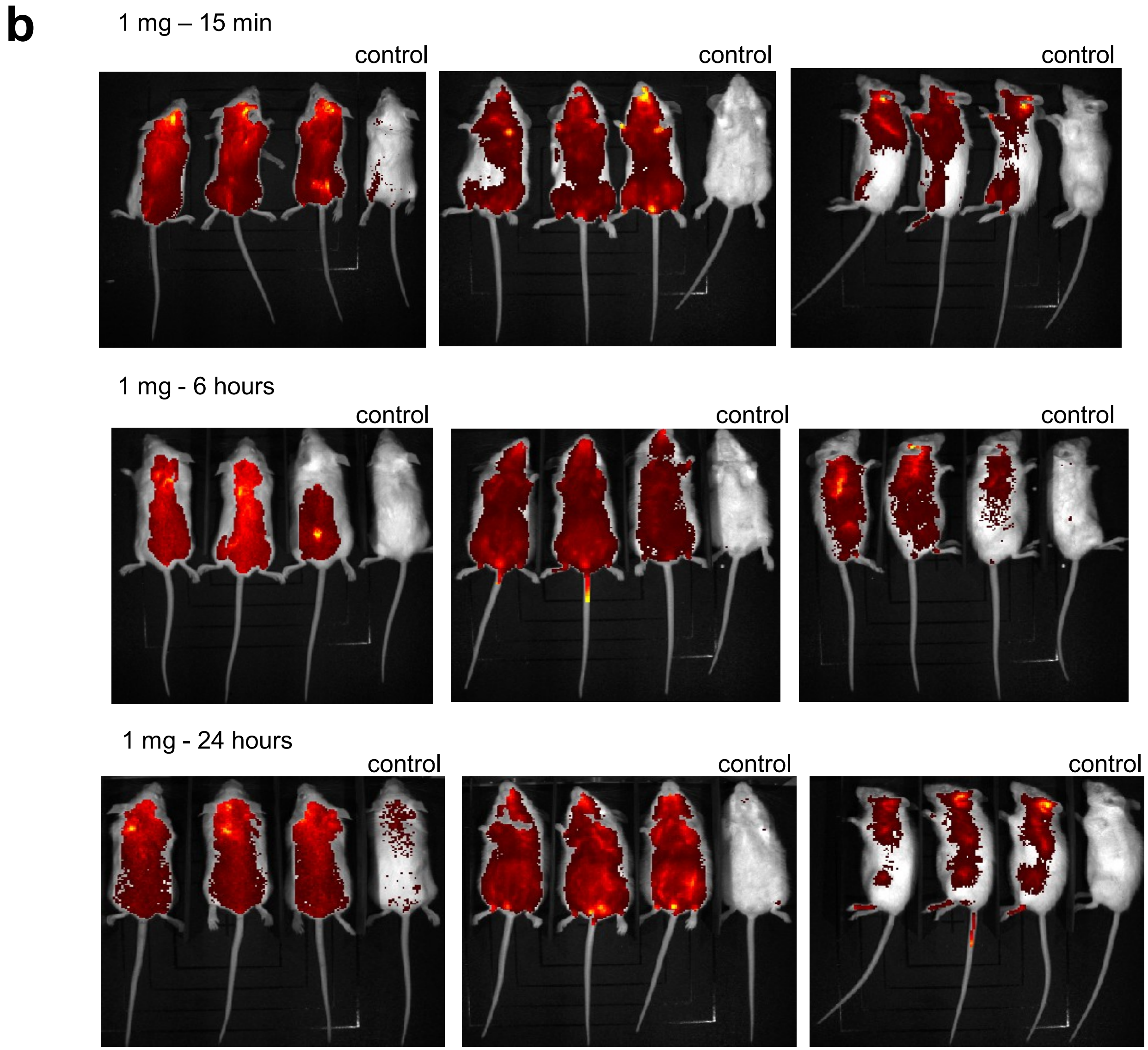

Supplementary Figure 4 (cont)

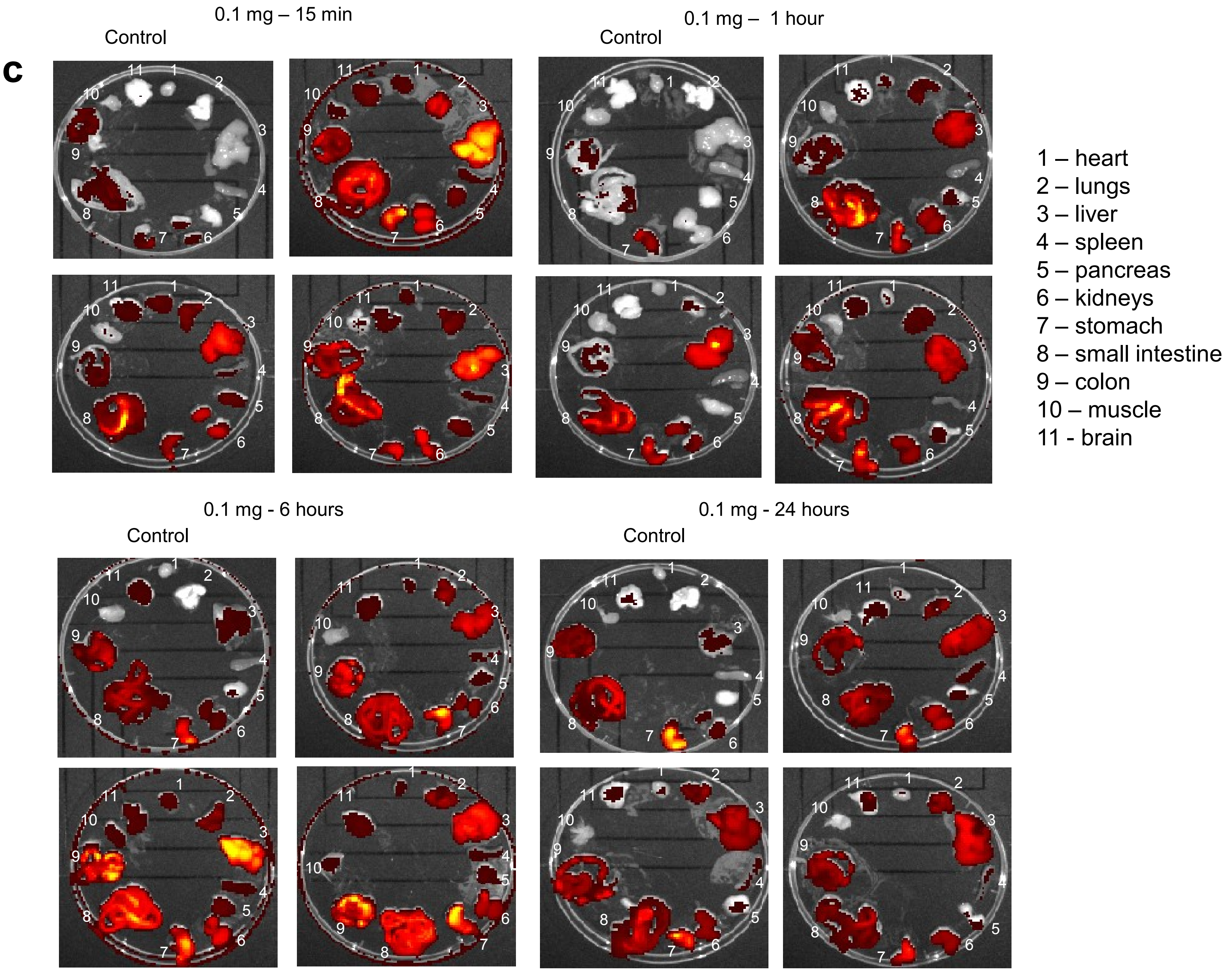

Supplementary Figure 4 (cont)

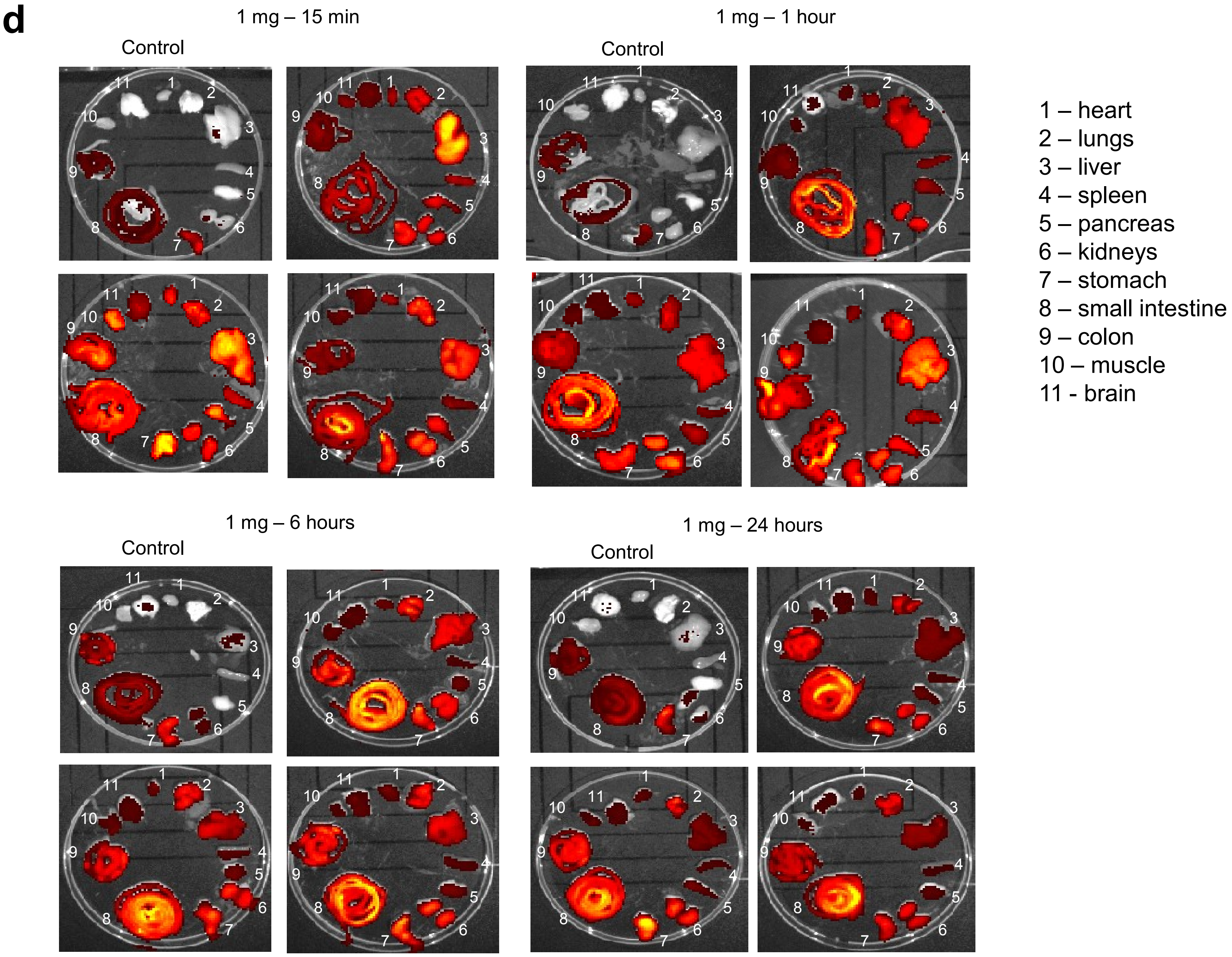

Supplementary Figure 4 (cont)

e

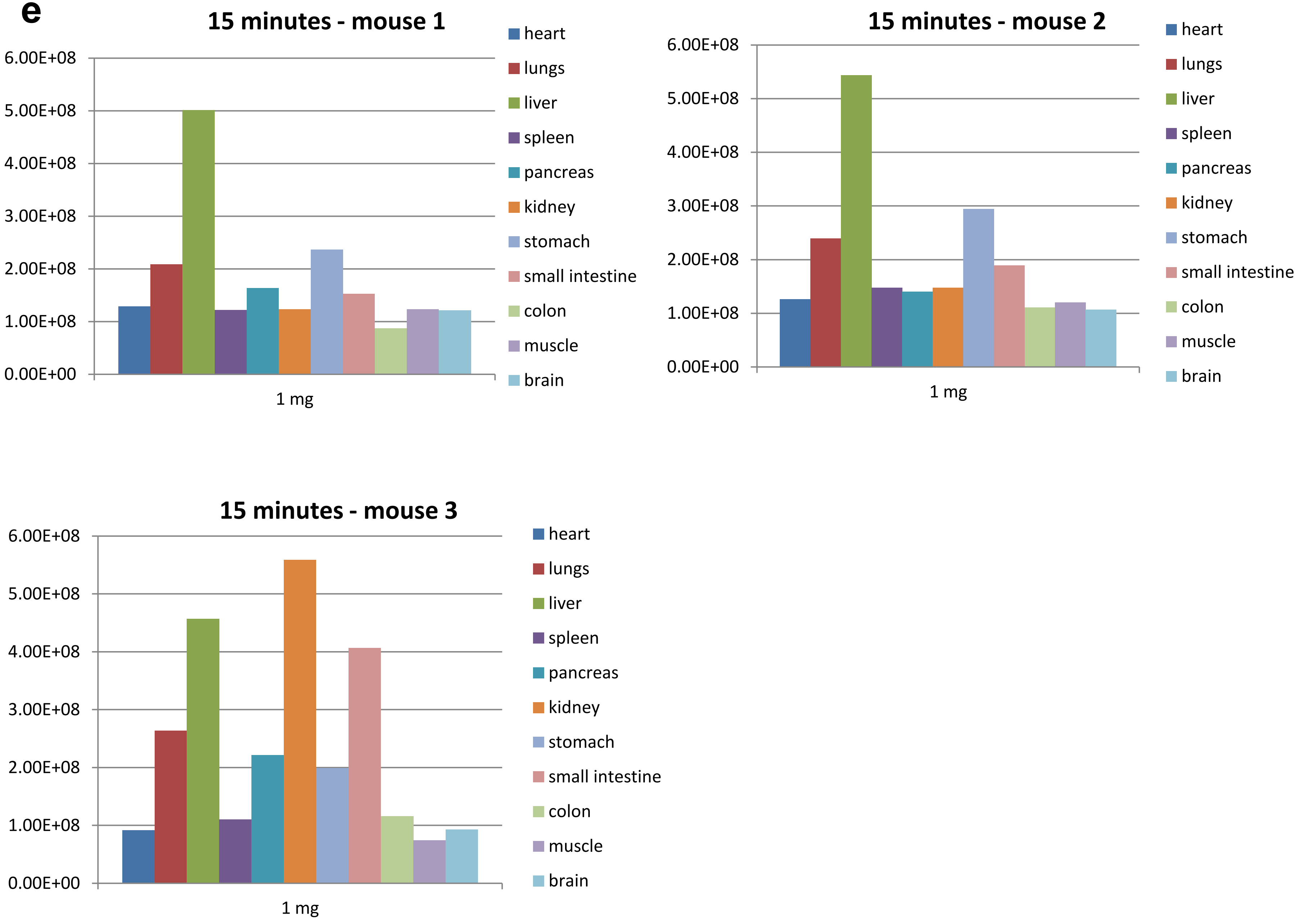

Supplementary Figure 4 (cont)

f

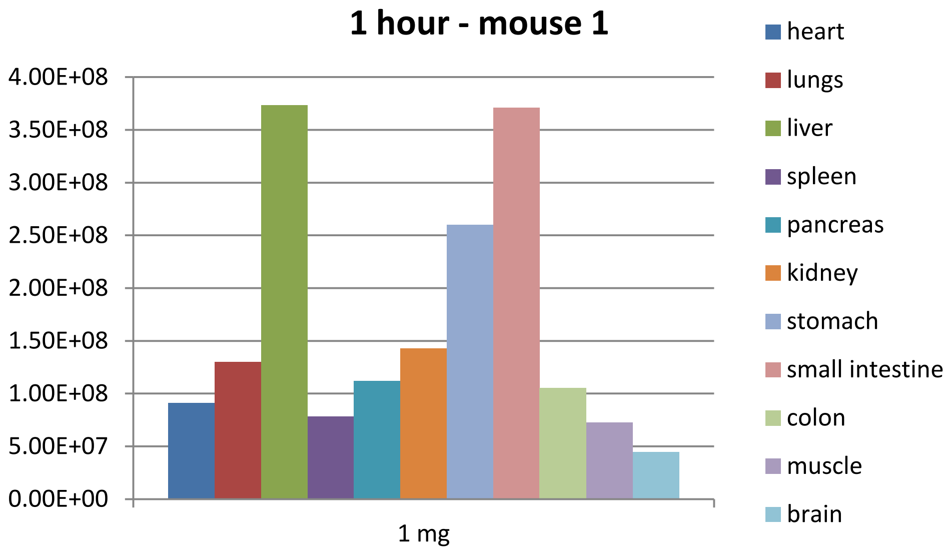

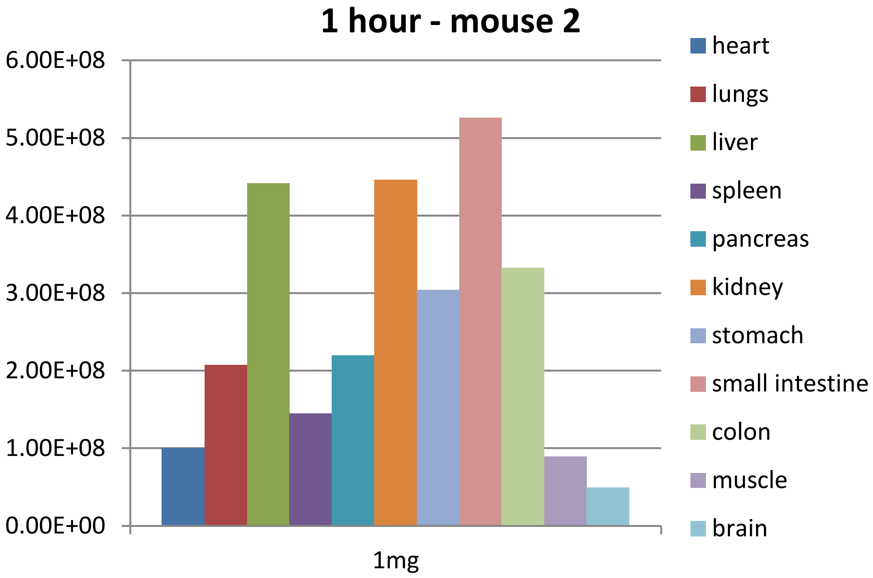

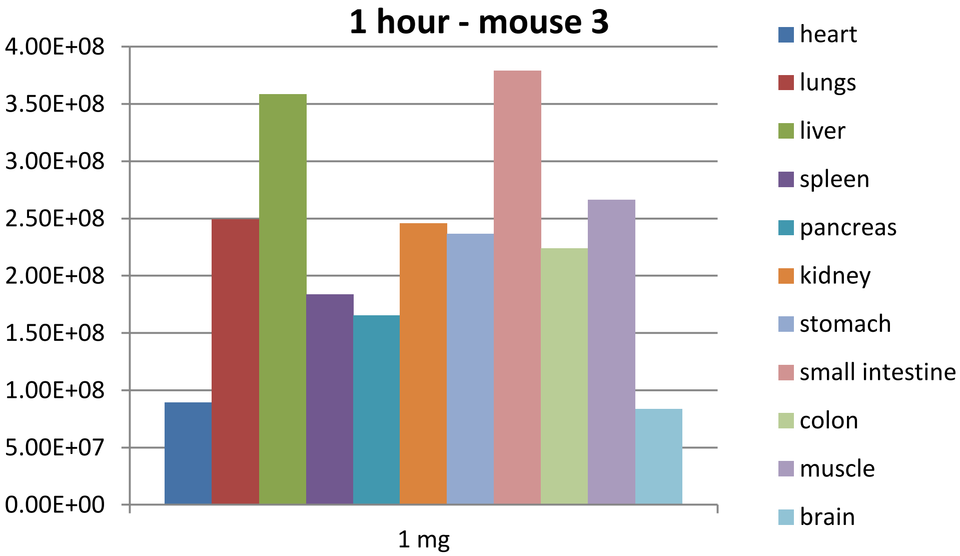

Supplementary Figure 4 (cont)

g

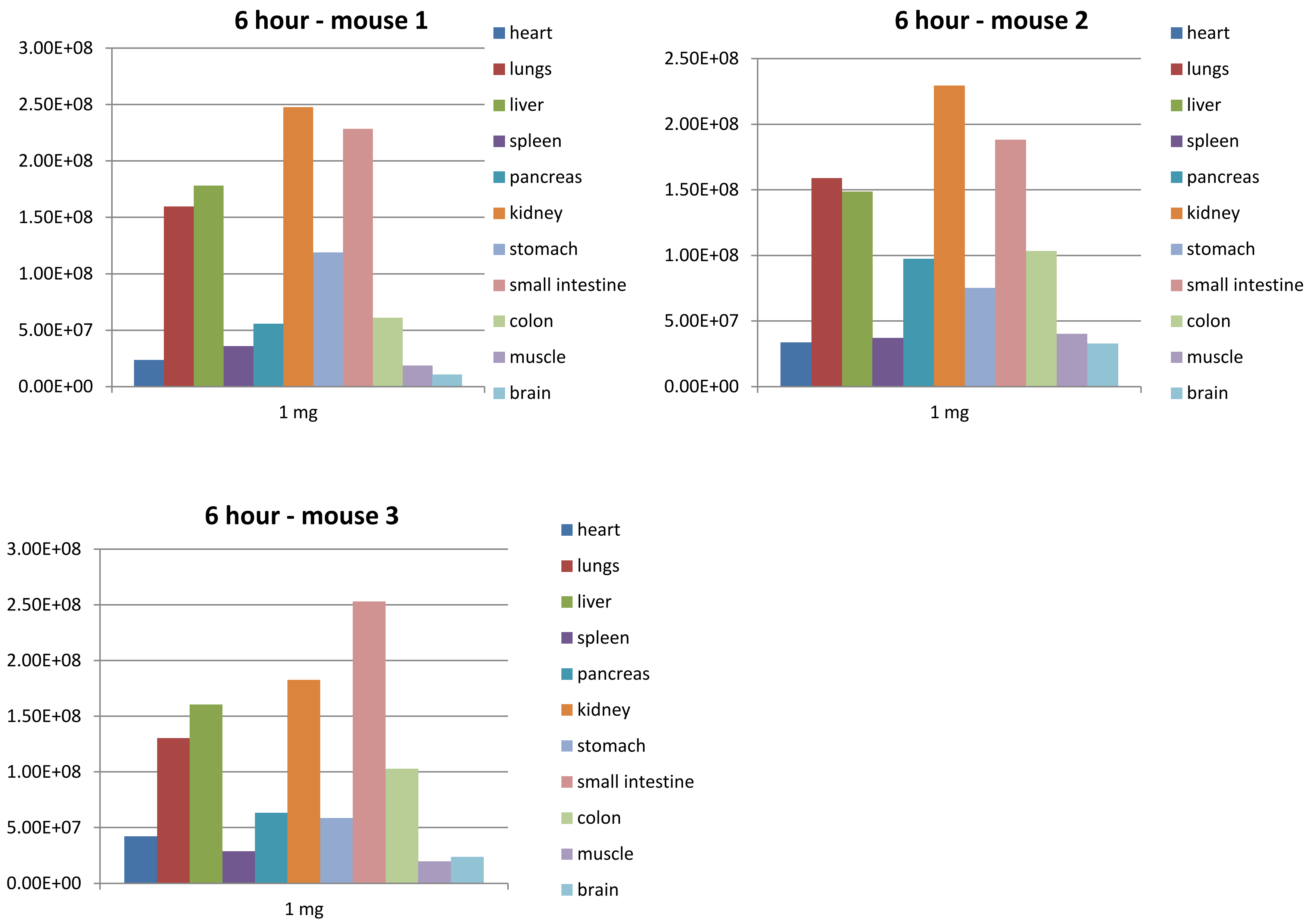

Supplementary Figure 4 (cont)

h

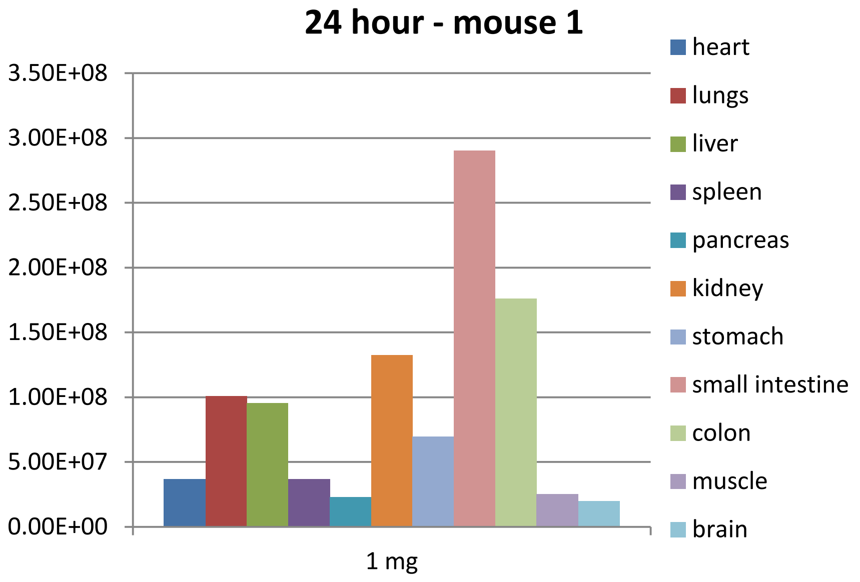

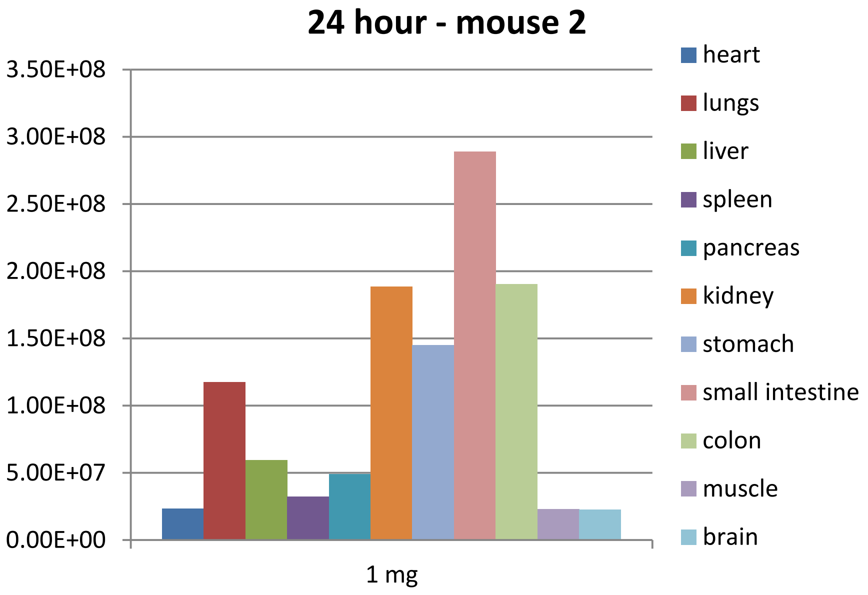

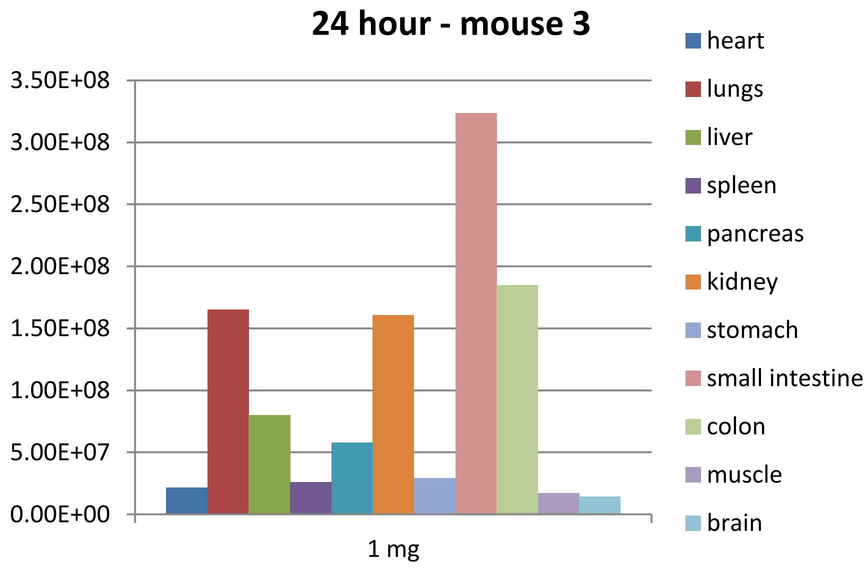

Supplementary Figure 4. Biodistribution of Cy7-labelled Xentry intravenously injected into mice. Mice were intravenously injected with 0.1 mg (a,c) and 1 mg (b,d) of Cy7-labelled Xentry, and the fluorescence of the whole bodies (a,b) and major organs (c,d) were recorded 30 min, 1, 6, and 24 h later using the Xenogen-IVIS® Kinetic-In Vivo Imaging System, and compared with untreated control littermates. Dorsal, ventral and side views of representative mice are illustrated. Refer to Figure 4d for the 1 hour time-point for whole body fluorescence of mice injected with 1 mg of Xentry. (e-h) Quantification of the fluorescence signal from the organs (d) of mice intravenously injected with 1 mg of Cy7-labelled Xentry.

Supplementary Figure 5

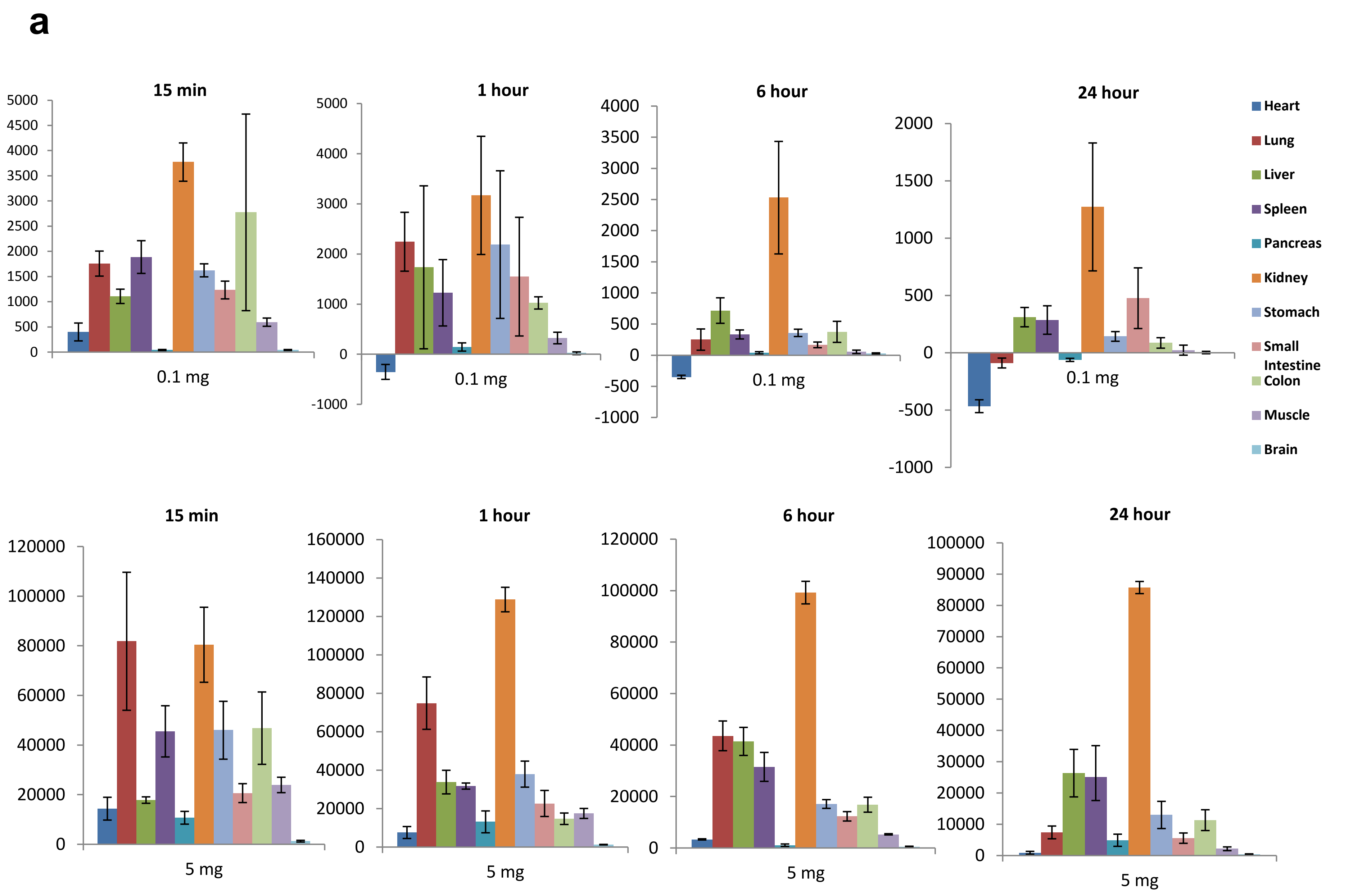

Supplementary Figure 5 (cont)

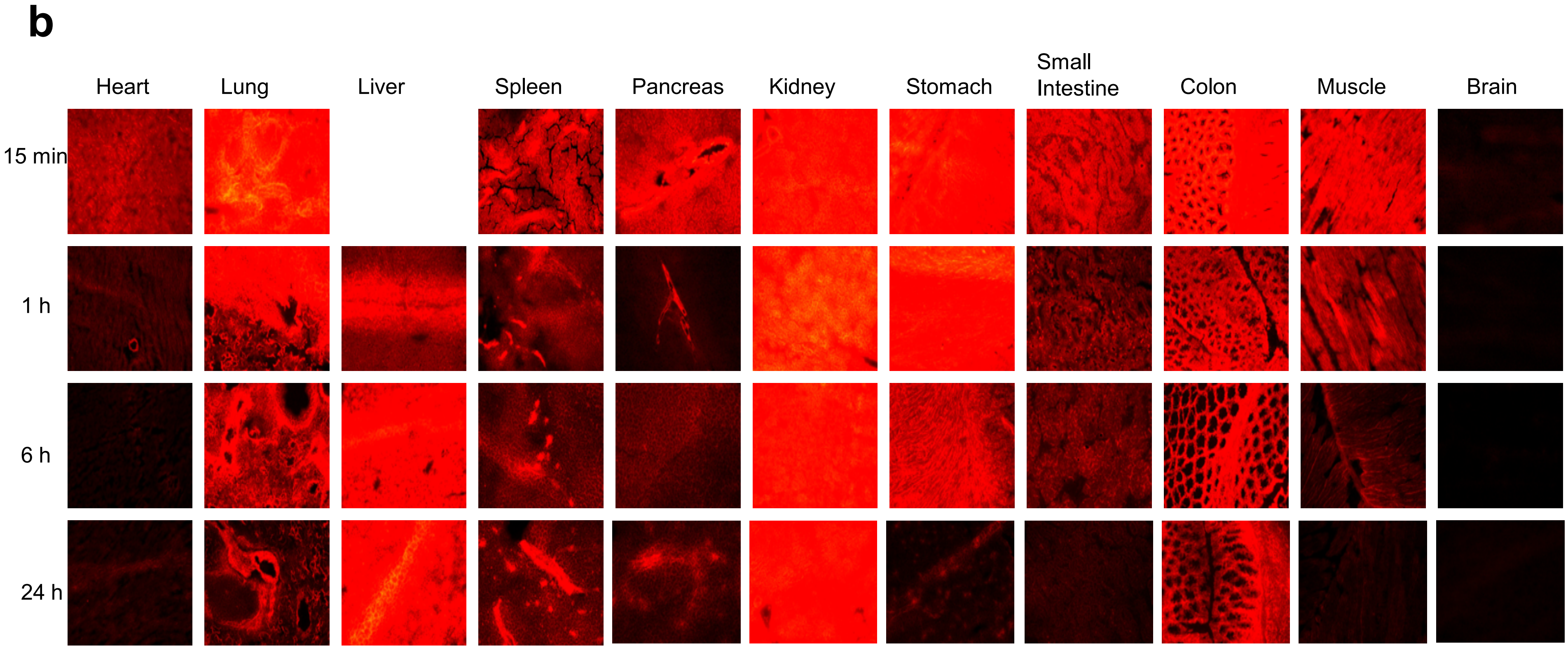

Supplementary Figure 5. Biodistribution of TAMRA-labelled Xentry intravenously injected into mice. (a) Mice were intravenously injected with 0.1 mg and 5 mg of TAMRA-labelled Xentry and the fluorescence of organ homogenates was measured 15 min, 1, 6, and 24 h later. Results are presented as the mean $\pm$ SD (n = 3). Refer to Figure 4d for the fluorescence of organ homogenates of mice injected with 1 mg of Xentry. (b) Fluorescence of sections of the major organs of mice intravenously injected with 5 mg of TAMRA-labelled Xentry. The liver sections for the 15 min time-point were lost.

Supplementary Figure 6

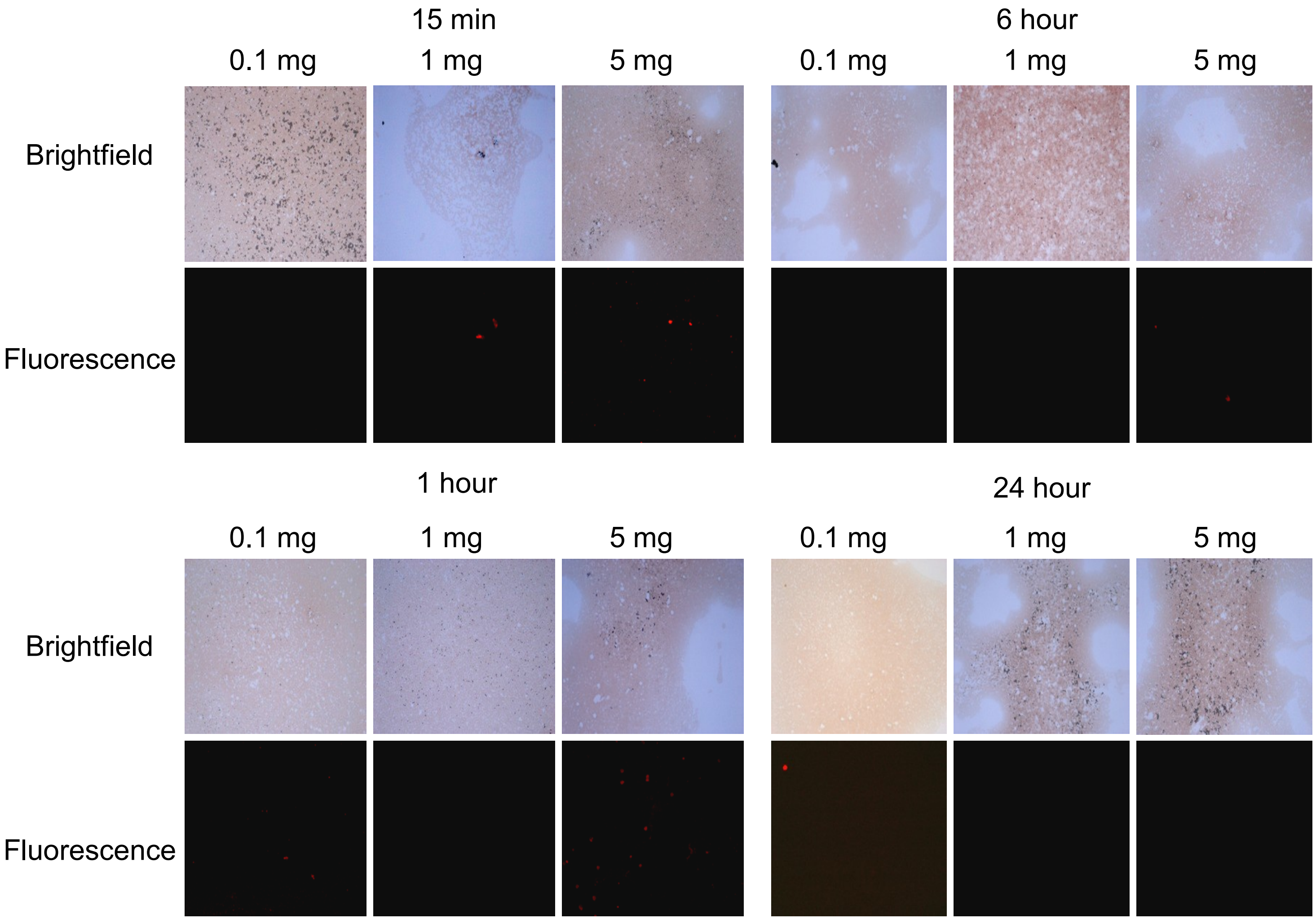

Supplementary Figure 6. Intravenously injected Xentry is not taken up and sequestered by blood cells. A sample of cardiac blood was collected from mice injected with 0.1, 1 and 5 mg of TAMRA-labelled Xentry at 4 different time points, and cytospun onto a slide. The blood sample was examined by brightfield and fluorescence microscopy.
